# Antigen-presenting ILC3 regulate T cell–dependent IgA responses to colonic mucosal bacteria

**DOI:** 10.1084/jem.20180871

**Published:** 2019-02-27

**Authors:** Felipe Melo-Gonzalez, Hana Kammoun, Elza Evren, Emma E. Dutton, Markella Papadopoulou, Barry M. Bradford, Ceylan Tanes, Fahmina Fardus-Reid, Jonathan R. Swann, Kyle Bittinger, Neil A. Mabbott, Bruce A. Vallance, Tim Willinger, David R. Withers, Matthew R. Hepworth

**Affiliations:** 1 Lydia Becker Institute of Immunology and Inflammation, University of Manchester, Manchester, UK; 2 Manchester Collaborative Centre for Inflammation Research, Division of Infection, Immunity and Respiratory Medicine, School of Biological Sciences, Manchester Academic Health Science Centre, University of Manchester, Manchester, UK; 3 Center for Infectious Medicine, Department of Medicine, Karolinska Institutet, Stockholm, Sweden; 4 Institute of Immunology and Immunotherapy (III), College of Medical and Dental Sciences, University of Birmingham, Birmingham, UK; 5 The Roslin Institute and Royal (Dick) School of Veterinary Sciences, University of Edinburgh, Easter Bush, UK; 6 Division of Gastroenterology, Hepatology, and Nutrition, The Children's Hospital of Philadelphia, Philadelphia, PA; 7 Division of Integrative Systems Medicine and Digestive Diseases, Imperial College London, South Kensington, UK; 8 Department of Pediatrics, British Columbia Children's Hospital, University of British Columbia, Vancouver, Canada

## Abstract

Intestinal immune homeostasis is dependent upon tightly regulated and dynamic host interactions with the commensal microbiota. Immunoglobulin A (IgA) produced by mucosal B cells dictates the composition of commensal bacteria residing within the intestine. While emerging evidence suggests the majority of IgA is produced innately and may be polyreactive, mucosal-dwelling species can also elicit IgA via T cell–dependent mechanisms. However, the mechanisms that modulate the magnitude and quality of T cell–dependent IgA responses remain incompletely understood. Here we demonstrate that group 3 innate lymphoid cells (ILC3) regulate steady state interactions between T follicular helper cells (TfH) and B cells to limit mucosal IgA responses. ILC3 used conserved migratory cues to establish residence within the interfollicular regions of the intestinal draining lymph nodes, where they act to limit TfH responses and B cell class switching through antigen presentation. The absence of ILC3-intrinsic antigen presentation resulted in increased and selective IgA coating of bacteria residing within the colonic mucosa. Together these findings implicate lymph node resident, antigen-presenting ILC3 as a critical regulatory checkpoint in the generation of T cell–dependent colonic IgA and suggest ILC3 act to maintain tissue homeostasis and mutualism with the mucosal-dwelling commensal microbiota.

## Abstract (precis)

ILCs are critical regulators of intestinal immune homeostasis and interactions with commensal bacteria. Melo-Gonzalez et al. show that ILC3 localize at the T–B cell interface of the colon-draining lymph node and interact with T follicular helper cells to limit mucosal IgA responses against commensal and pathogenic bacteria.

## Graphical Abstract

**Figure:**
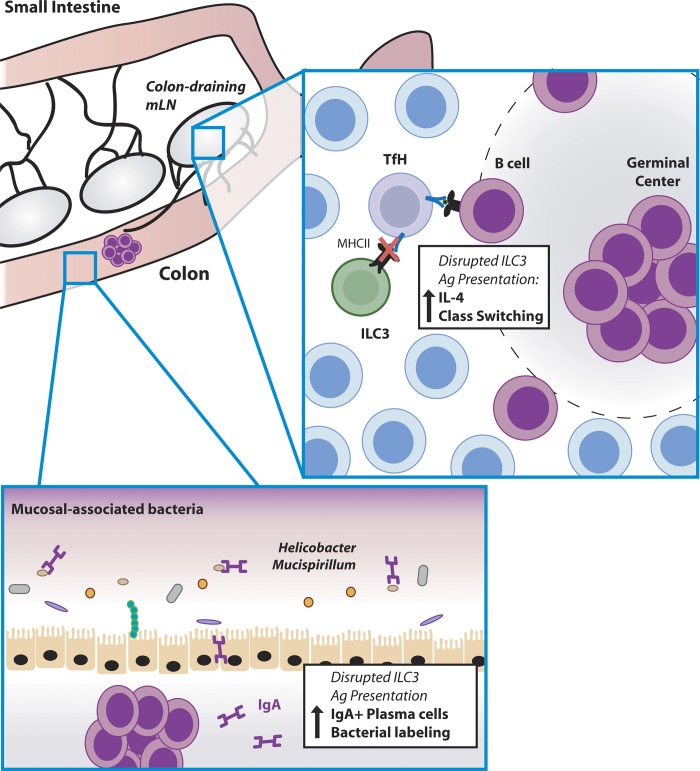

## Introduction

Homeostatic colonization of the gastrointestinal tract by the commensal microbiota is increasingly appreciated to modulate a wide range of basic biological processes including behavior, pathogen colonization, nutrient uptake, and immune development (Hooper et al., 2012; Belkaid and Hand, 2014; Honda and Littman, 2016). In contrast, dysregulated responses toward commensal bacteria, or shifts in the composition of the intestinal microbiota that favor the outgrowth of opportunistic bacterial pathobionts, have been associated with disease pathology in a wide range of conditions, including inflammatory bowel disease. As such, host interactions with the intestinal microbiota are tightly regulated to maintain tissue health and homeostasis. This is in part achieved via physical segregation of the vast majority of commensal microbiota from the underlying tissue by the production of highly organized mucus layers, which are rich in antimicrobial peptides, and through the maintenance of epithelial barrier integrity to prevent bacterial translocation (Hooper et al., 2012; Belkaid and Hand, 2014; Honda and Littman, 2016). Nonetheless, physical segregation of commensal microbes is not absolute, and some commensal species have adapted to thrive within the mucosal layer or epithelial niche, yet are tolerated under homeostatic circumstances and do not elicit inflammation in the healthy intestine (Honda and Littman, 2016). However, the underlying mechanisms for this phenomenon remain incompletely understood.

Tolerance toward the commensal microbiota is further maintained by the intestinal immune system. A broad range of immune-mediated mechanisms have coevolved to cooperatively suppress inflammatory responses against otherwise beneficial commensal microbes and to prevent inflammation in the gastrointestinal tract. Among these the production of mucosal antibodies, particularly IgA, by tissue-resident B cells is key to controlling the composition of the intestinal microbiota (Macpherson et al., 2015; Kubinak and Round, 2016). IgA acts by excluding bacterial access to the underlying tissue by neutralizing bacterial toxins and through agglutination or enchained growth of targeted bacterial species—which together act to reduce colonization and increase shedding in the feces (Macpherson et al., 2015; Kubinak and Round, 2016; Moor et al., 2017). Conversely, IgA can also help to promote mutualism by selecting for communities of bacteria with beneficial properties (Fagarasan et al., 2010).

IgA can be generated via distinct mechanisms, either in a T cell–independent manner or via coordinated interactions with T follicular helper cells (TfH) in lymphoid tissues that select for high-affinity B cell clones, and promotes class switching within germinal centers (GCs). However, the mechanisms that control the magnitude and quality of IgA responses to commensal bacterial species are incompletely understood. Recent studies have indicated the majority of IgA produced at steady state is produced in a T cell–independent manner and secreted within the small intestine, rather than the colon where the microbial load is highest (Bunker et al., 2015). Moreover, the vast majority of the small intestinal IgA repertoire appears to be polyreactive and is present even in the absence of the microbiota (Bunker et al., 2017). In contrast, under homeostatic conditions only a small subset of commensal bacterial species elicit T cell–dependent IgA responses and exhibit a relatively enhanced level of IgA coating (Palm et al., 2014; Bunker et al., 2015). The reasons why some bacterial species preferentially trigger a T cell–dependent, high-affinity IgA response under homeostatic conditions is unclear; however, emerging evidence suggests these bacterial species may be preferentially localized within relatively immunostimulatory niches such as the mucus layer or in close contact with the intestinal epithelium (Palm et al., 2014; Bunker et al., 2015). These bacterial populations have been suggested to have an increased propensity to drive colitis when intestinal homeostasis is perturbed, and thus their residence within the gut must be tightly controlled by the immune system. In contrast, high-affinity IgA has been suggested to support mutualism by supporting, refining, and maintaining commensal bacterial communities, and colonization of mucosal-dwelling species has also been suggested to provide mutualistic benefits for the host at homeostasis (Fagarasan et al., 2010; Sutherland et al., 2016; Campbell et al., 2018; Donaldson et al., 2018). Despite these advances, the mechanisms that act to regulate the quality and magnitude of T-dependent antibody responses toward commensal bacteria in the intestine remain poorly understood.

Innate lymphoid cells (ILCs) are a family of transcriptionally poised, tissue-resident effector cells with critical roles in intestinal immunity and inflammation (Klose and Artis, 2016). In particular, RORγt^+^ group 3 ILC (ILC3) are key regulators of intestinal homeostasis and promote host–microbiota mutualism through constitutive production of cytokines, such as IL-22, which acts on intestinal epithelial cells to reinforce barrier integrity and to induce epithelial cell fucosylation and antimicrobial peptide secretion (Sonnenberg and Artis, 2012; Klose and Artis, 2016). In addition to their role as effector cells, ILC3 are increasingly appreciated to have broader immunoregulatory functions via interactions with the adaptive immune system (Hepworth and Sonnenberg, 2014; Melo-Gonzalez and Hepworth, 2017). In particular, ILC3 help to maintain tissue-resident regulatory T cell (T reg) populations through GM-CSF–mediated effects on tolerogenic myeloid populations (Mortha et al., 2014). Moreover, CCR6-expressing lymphoid tissue inducer (LTi)–like ILC3 highly express MHCII and exhibit antigen-presenting function and have been demonstrated to directly suppress inflammatory effector CD4^+^ T cell responses toward commensal antigens in the intestinal-draining mesenteric lymph node (mLN; Hepworth et al., 2013, 2015). LTi-like ILC3 also selectively localize within the mLN at the interface between T cells and B cells (Mackley et al., 2015), provoking the hypothesis that ILC3 have roles in regulating the generation of antibody responses within the gastrointestinal tract and associated lymphoid tissue. In this regard, ILC3 have previously been demonstrated to modulate T cell–independent B cell responses and antibody production via the production of lymphotoxin and B cell survival factors such as BAFF and APRIL (Kruglov et al., 2013; Magri et al., 2014; Reboldi et al., 2016). However, whether ILC3 populations also orchestrate T cell–dependent IgA responses toward the commensal microbiota is not known.

In this study, we demonstrate that ILC3 colocalization within the interfollicular regions of the mLN facilitates MHCII-dependent regulation of TfH responses and limits GC reactions. Presentation of antigen by ILC3 to TfH was required to suppress the subsequent generation of colonic IgA^+^ B cell responses against both commensal and pathogenic bacterial species residing within the mucosa of the large intestine. Together, these studies suggest ILC3 act as an important checkpoint in the generation of T cell–dependent, antigen-specific IgA responses to commensal microbes in the colon under homeostatic conditions. ILC3 regulation of colonic IgA may play a critical role in maintaining tissue homeostasis by controlling the mutualism with commensal species that establish residence in the host mucosal niche.

## Results and discussion

### ILC3 establish residence within the interfollicular region of the mLN and interact with TfH cells through sensing of migratory cues

**Figure 1.**
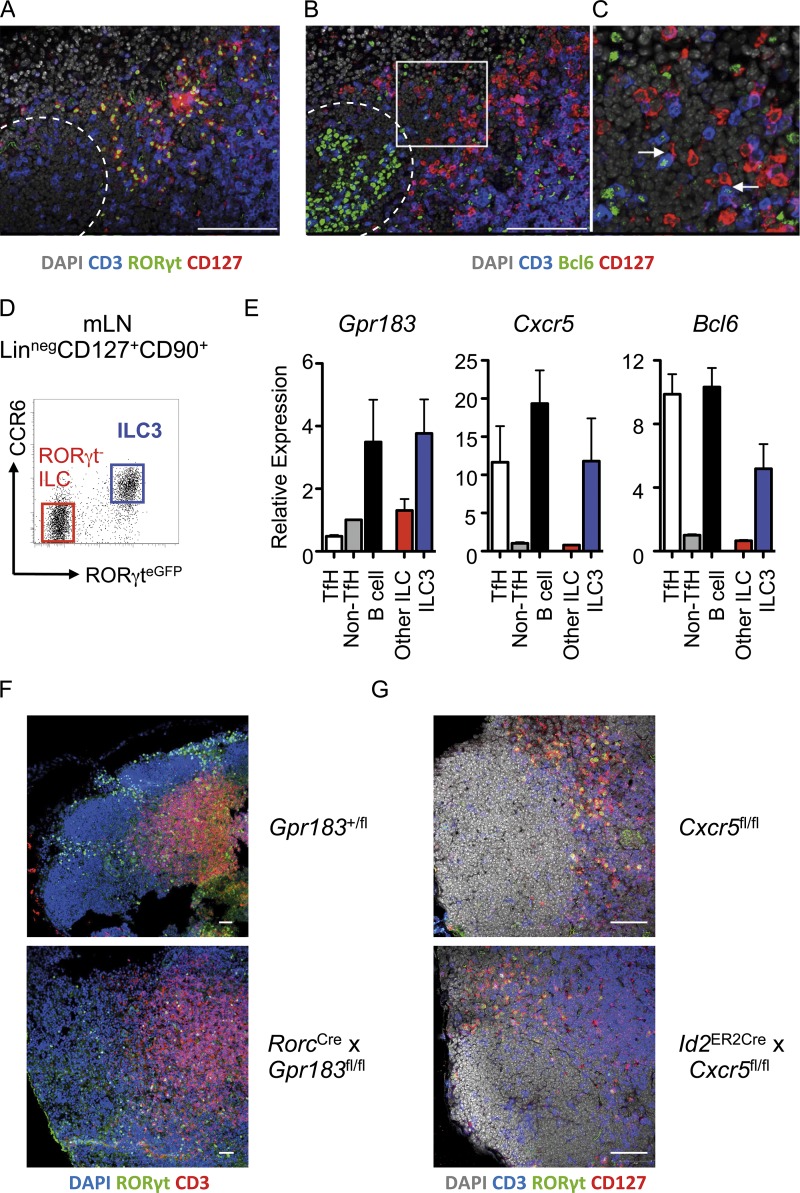
**LTi-like ILC3 localize to interfollicular regions of the mLN and interact with TfH cells. (A and B)** Serial sections of mLN demonstrating (A) positioning of RORγt^+^CD127^+^ CD3^−^ ILC3 and (B) colocalization of CD127^+^CD3^−^ ILC with CD127^−^CD3^+^Bcl6^+^ TfH cells in wild-type C57BL/6 mice (*n* = 6), representative of two independent experiments. Dotted line indicates follicular border. Bars, 100 µm. **(C)** Higher magnification image of insert indicated in B. Arrows indicate contacts between ILC3 and TfH cells in interfollicular region. **(D and E)** (Live CD45^+^/Lin^neg^/CD127^+^) RORγt^eGFP+^ LTi-like ILC3 (*n* = 3; blue) and RORγt-negative ILC (*n* = 3; red) were sorted from the mLN of RORγt^eGFP^ mice along with (CD3^−^CD4^+^) PD-1^+^ICOS^+^ TfH (*n* = 6), PD-1^−^ICOS^−^ conventional T cells (*n* = 6), and CD19^+^B220^+^MHCII^+^ B cells (*n* = 6; D) and assessed for expression of *Gpr183*, *Cxcr5*, and *Bcl6* (E), with data representative of two independent experiments. **(F)** Localization of DAPI^+^RORγt^+^ CD3^−^ ILC3 in the mLN of *Gpr183*^+/fl^ control mice or *Rorc*^Cre^ × *Gpr183*^fl/fl^ mice (*n* = 5 per group), representative of two independent experiments. **(G)** Localization of CD127^+^RORγt^+^CD3^−^ ILC3 in the mLN of *Cxcr5*^fl/fl^ control mice (*n* = 6) or *Id2*^CreERT2^ × *Cxcr5*^fl/fl^ mice (*n* = 7) treated four times with tamoxifen and rested for 1 mo, representative of two independent experiments. Bar, 50 µm. All data shown as mean ± SEM.

ILC3 have emerging roles in the regulation of adaptive immunity and have been shown to localize at the boundary between the T cell zone and B cell follicles within lymph nodes (Mackley et al., 2015). However, the functional importance of this localization and the molecular machinery required for positioning of ILC3 within this lymph node niche remain incompletely defined. The interfollicular regions of lymphoid tissues are critical sites for initial interactions between specialized TfH cells and cognate B cells that ultimately result in GC reactions and the production of high-affinity antibody (Kerfoot et al., 2011; Vinuesa and Cyster, 2011). Analysis of ILC3 localization using serial sections indicated that CD3^−^RORγt^+^CD127^+^ ILC3 colocalized and formed frequent contacts with CD3^+^Bcl6^+^ T cells in the interfollicular region, at the border of B cell follicles also containing CD3^−^Bcl6^+^ GC B cells (Fig. 1, A–C; and Fig. S1 A). Interactions between TfH and B cells in this lymph node niche are elegantly orchestrated by transcription factors, local migratory gradients, and tightly regulated expression of a range of chemokine receptors (Vinuesa and Cyster, 2011). Thus, we hypothesized that ILC3 localization within the interfollicular niche may be dictated through similar pathways. To test this hypothesis, CCR6^+^RORγt^+^ LTi-like ILC3 were sort-purified from the mLN of RORγt^eGFP^ reporter mice alongside RORγt^−^ ILCs, TfH, and B cells and assessed for migratory receptors that are known to facilitate T–B cell interactions (Fig. 1, D and E). Lymph node ILC3 were found to express G protein–coupled receptor 183 (*Gpr183*, also known as EBI2), as recently described (Chu et al., 2018; Emgård et al., 2018), as well as *Cxcr5*—at levels similar to B cells and TfH, respectively (Fig. 1 E and Fig. S1 B). In addition, ILC3 expressed moderate levels of the transcription factor *Bcl6* at the mRNA level (Fig. 1 E), although we were unable to consistently detect Bcl6 protein in ILC3 (data not shown). Together, these data indicate that LTi-like ILC3 may use conserved molecular machinery to establish residence at interfollicular sites within the lymph node, and that they may be ideally located to modulate TfH and potentially influence downstream induction of B cell responses and antibody production.

To determine the relative contribution of ILC3-expressed migratory receptors, we examined ILC3 localization in the lymph nodes of mice lacking *Gpr183*, *Cxcr5*, or *Ccr6*—the last a defining surface marker of LTi-like ILC3 and central regulator of follicular B cell dynamics and GC reactions (Reimer et al., 2017). *Gpr183* directs the localization of lymphocytes through sensing of tightly regulated gradients of its oxysterol ligand 7α,25-hydroxycholesterol, and has recently been demonstrated to be required for ILC3 migration to intestinal tissue lymphoid structures—including cryptopatches and isolated lymphoid follicles (Emgård et al., 2018). To investigate the role of *Gpr183* in ILC3 localization further, we visualized ILC3 in the mLN of *Rorc*^Cre^ × *Gpr183*^fl/fl^ mice, where *Gpr183* expression is ablated in ILC3 (and T cells). In contrast to wild-type controls, in which RORγt^+^ ILC3 formed distinct clusters within the interfollicular regions, *Gpr183*-deficient ILC3 failed to cluster and were instead dispersed throughout the lymph node (Fig. 1 F). *Gpr183* was not required for entry into the lymph node per se as ILC3 frequencies within the mLN of mice were unaffected by deletion of ILC3-intrinsic *Gpr183* (Fig. S1, C and D). Thus, in line with another recent study, our data suggest *Gpr183* is required for ILC3 localization to the interfollicular border of the mLN (Chu et al., 2018).

CXCR5 is a marker of TfH and orchestrates colocalization of TfH with follicular B cells. In addition, CXCR5 has been described as a marker of bona fide fetal LTi cells and is important for the establishment of normal lymph node architecture (Finke et al., 2002; Ohl et al., 2003). To test the role of this chemokine receptor in ILC3 positioning within the adult lymph node, we generated *Id2*^CreERT2^ ROSA^RFP^ × *Cxcr5*^flox/flox^ mice (hereafter referred to as *Cxcr5*^ΔILC^), which allow for the inducible deletion of CXCR5 in ID2-expressing ILC and thus circumvent any potential developmental effects on lymphoid tissue formation. RFP^+^ ILC3 from tamoxifen-treated *Cxcr5*^ΔILC^ mice were confirmed to have effective gene deletion in comparison to RFP^+^ ILC3 from CXCR5^+/+^ controls (Fig. S1 E). However, immunofluorescence imaging of ILC3 revealed that CXCR5 was not absolutely required to maintain ILC3 residency in the interfollicular region once established (Fig. 1 G). Similarly, CCR6 was also dispensable for ILC3 localization within the interfollicular niche of the mLN (Fig. S1 F). Together these findings indicate lymph node–resident LTi-like ILC3 use sensing of cholesterol ligand cues via *Gpr183* to establish residence within the interfollicular border where they interact with TfH.

### Interfollicular LTi-like ILC3 limit homeostatic TfH and B cell responses and colonic IgA

**Figure 2.**
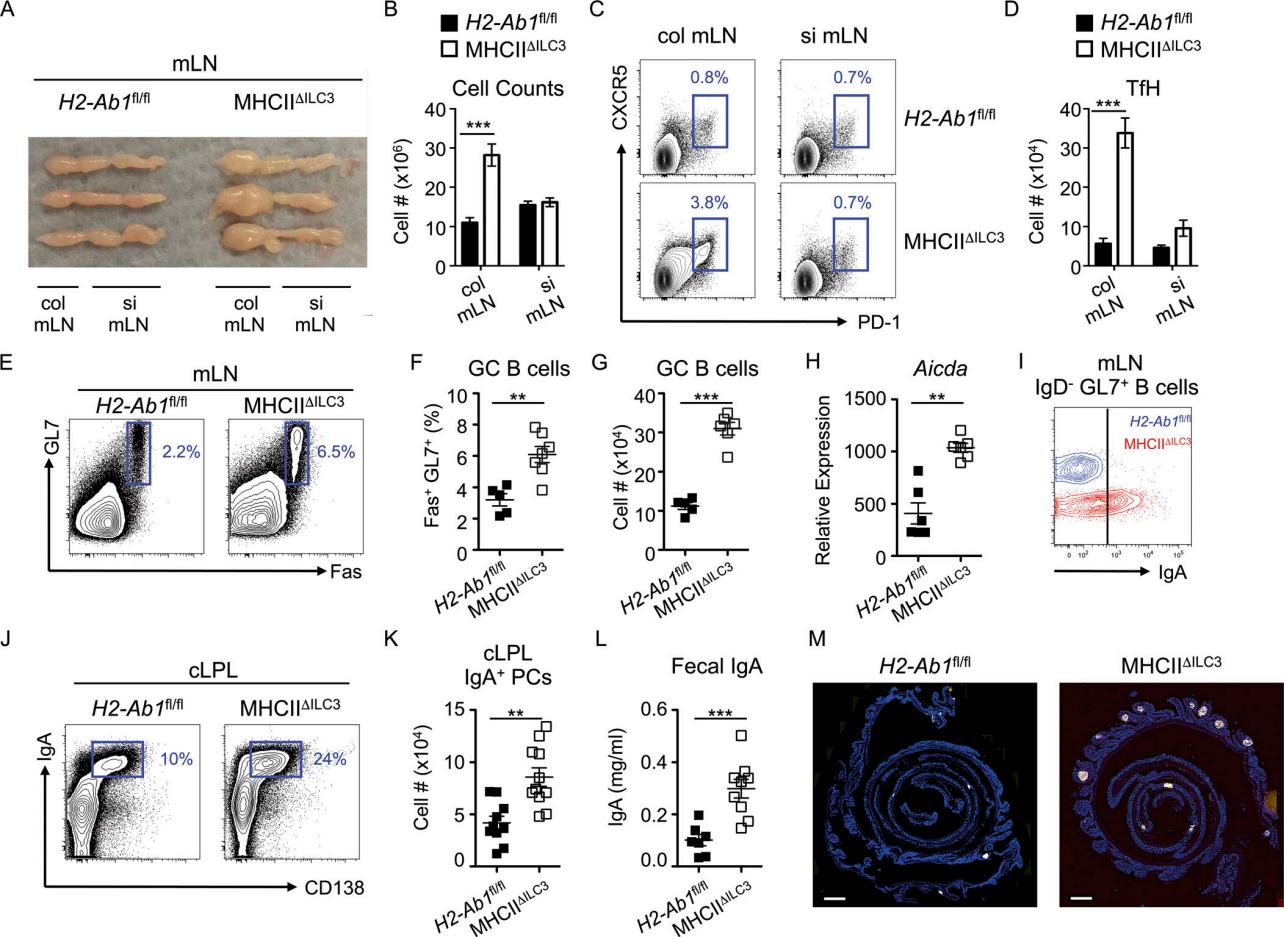
**Antigen-presenting LTi-like ILC3 limit the colon-associated TfH–B cell axis and IgA production. (A and B)** Images of mLN chains with colon (col) and small intestinal (si) draining lymph nodes indicated (A) and cell counts of colonic or small intestinal draining nodes isolated from *Rorc*^Cre^ x *H2-Ab1*^fl/fl^ mice (MHCII^ΔILC3^; *n* = 14) and *H2-Ab1^fl/fl^* littermate controls (*n* = 5; B), representative of at least three independent experiments. **(C and D)** Representative flow cytometry plots (C) and cell numbers of (live CD45^+^/CD3^+^CD4^+^/CD25^−^GITR^−^)CXCR5^+^PD1^+^ TfH in colon and small intestinal draining lymph nodes isolated from MHCII^ΔILC3^ mice (*n* = 14) and *H2-Ab1^fl/fl^* littermate controls (*n* = 5; D), representative of at least three independent experiments. **(E–G)** Representative flow cytometry plots (E), cell frequencies (F), and cell numbers (G) of mLN (live CD45/CD3^−^MHCII^+^/B220^+^CD19^+^)GL7^+^Fas^+^ GC B cells isolated from MHCII^ΔILC3^ mice (*n* = 7) and *H2-Ab1^fl/fl^* littermate controls (*n* = 5), representative of eleven independent experiments. **(H)** Relative expression of *Aicda* by sort-purified B cells (*n* = 6), pooled from two independent experiments. **(I)** Representative surface staining of IgA from class-switched B cells from E–G. **(J and K)** Representative flow cytometry plots (J) and cell numbers of (live CD45^+^/CD3^−^MHCII^+^)IgA^+^CD138^+^ plasma cells in the colonic lamina propria (cLPL) isolated from MHCII^ΔILC3^ mice (*n* = 11) and *H2-Ab1^fl/fl^* littermate controls (*n* = 10; K); data are pooled from two experiments and representative of four independent experiments in total. **(L)** Concentration of free IgA measured by ELISA in feces isolated from MHCII^ΔILC3^ mice (*n* = 11) and *H2-Ab1^fl/fl^* littermate controls (*n* = 7) and representative of three independent experiments. **(M)** Histological analysis of B220^+^ cells in colonic swiss rolls isolated from MHCII^ΔILC3^ mice and *H2-Ab1^fl/fl^* littermate controls (*n* = 4–5 per group), with three independent experiments. Bars, 1,000 µm. All data shown as mean ± SEM and analyzed by Mann–Whitney *U* test; **P ≤ 0.01; ***P ≤ 0.001.

The observation that ILC3 are found in contact with TfH populations provoked the possibility that ILC3 may modulate T-dependent B cell responses in the intestinal-draining lymph node. We have previously demonstrated that one major mechanism through which LTi-like ILC3 exert their effects within the mLN is through the presentation of antigen to CD4^+^ T cells via MHCII, and we confirmed MHCII expression on interfollicular-resident ILC3 by immunofluorescence and by flow cytometry of CCR6^+^ LTi-like ILC3 in intestinal tissue and mLN (Fig. S1, G–M; Hepworth et al., 2013, 2015). Given the close cellular interactions between ILC3 and TfH within the interfollicular regions, we hypothesized that steady state TfH responses may be controlled by ILC3-dependent antigen presentation. To test this hypothesis, we quantified TfH in mice that lack ILC3-intrinsic MHCII expression (MHCII^ΔILC3^) or in *H2-Ab1*^fl/fl^ littermate controls in which antigen-presenting function is intact (Hepworth et al., 2013, 2015). Notably, 10–12-wk-old mice lacking ILC3-intrinsic MHCII expression exhibited elevated frequencies of (CD3^+^CD4^+^)CXCR5^+^PD-1^+^ TfH at steady state relative to littermate control mice (Fig. S2, A and B). This increase was specific to Bcl6^+^FoxP3^−^ TfH cells since no difference was observed the numbers of regulatory FoxP3^+^Bcl6^+^ follicular T cells (TfR; Fig. S2, C–E). These data indicate ILC3 may directly limit TfH responses at steady state through antigen presentation, independently of changes in the regulatory TfR cell population. The mLN consists of a series of discrete individual nodes, which have recently been demonstrated to differentially drain specific regions of the gastrointestinal tract, and profiling of ILC subsets within these individual nodes suggested MHCII^+^ ILC3 may be relatively enriched specifically in the colon-draining lymph node of the mLN (Fig. S1, H–J; Houston et al., 2016). Strikingly, we observed a selective lymphadenopathy in MHCII^ΔILC3^ mice in the first node of the mLN chain, proximal to the cecum, which has been demonstrated to be the major colon-draining node of the mLN (Houston et al., 2016; Fig. 2, A and B). In comparison, the small intestinal draining nodes remained largely unchanged in size and cellularity in the absence of ILC3-intrinsic antigen presentation (Fig. 2, A and B). Comparison of TfH frequencies and cell numbers in the colon-draining or small intestine–draining nodes of the mLN revealed increased TfH responses that were predominantly restricted to the colon-draining node of the mLN of MHCII^ΔILC3^ mice (Fig. 2, C and D). Collectively, these data suggest that MHCII^+^ ILC3 may largely act to restrict TfH responses initiated by antigen derived from the colon rather than the small intestine.

In line with enhanced TfH activity, GC B cell responses were increased in the mLN of MHCII^ΔILC3^ mice as compared with controls (Fig. 2, E–G). Moreover, mLN-resident B cells from MHCII^ΔILC3^ mice had increased *Aicda* expression—indicative of somatic hypermutation and class switch recombination (Fig. 2 H). GC B cells in MHCII^ΔILC3^ mice demonstrated increased class switching toward both IgG1 (Fig. S2 F) and IgA (Fig. 2 I). Strikingly, we also observed a significant increase in the frequencies and numbers of IgA^+^ plasma cells in the colons of MHCII^ΔILC3^ mice (Fig. 2, J and K), which was associated with increased titers of fecal IgA (Fig. 2 L). Moreover, large B cell–containing lymphoid follicles were observed in the colon of MHCII^ΔILC3^ mice, but not in littermate controls (Fig. 2 M)—some of which also exhibited evidence of active GCs (Fig. S2, G and H). Notably, the lack of ILC3-intrinsic MHCII had no effect on TfH or B cell responses in the Peyer’s patches or the small intestinal lamina propria—the major intestinal site for the induction of T-independent IgA (Fig. S2, I–N). This further indicates that MHCII^+^ ILC3 selectively limit TfH-driven B cell responses and IgA production in the colon. Nonetheless, as ILC3 have also been demonstrated to modulate B cell responses and IgA production directly in a T-independent manner, we further determined whether loss of ILC3-intrinsic MHCII disrupted broader ILC3 functionality. However, ILC3 from MHCII^ΔILC3^ mice and littermate controls equally expressed surface lymphotoxin, *Tnfsf13* (APRIL) and *Dll1* (Fig. S2, O and P)—suggesting intrinsic antigen presentation rather than changes in the provision of B cell survival signals accounts for altered B cell responses in MHCII^ΔILC3^ mice. Additionally, we have previously demonstrated that MHCII-deficient ILC3 are not defective in their ability to produce IL-22 (Hepworth et al., 2013), and absence of ILC3-intrinsic MHCII was not associated with dysregulated intestinal barrier function (Fig. S2 Q). Our data therefore indicate mLN-resident ILC3 limit TfH responses and mucosal antibody production in the colon in the presence of an intact intestinal barrier.

### IgA generated in the absence of ILC3 antigen presentation targets colonic mucosa-resident bacteria

**Figure 3.**
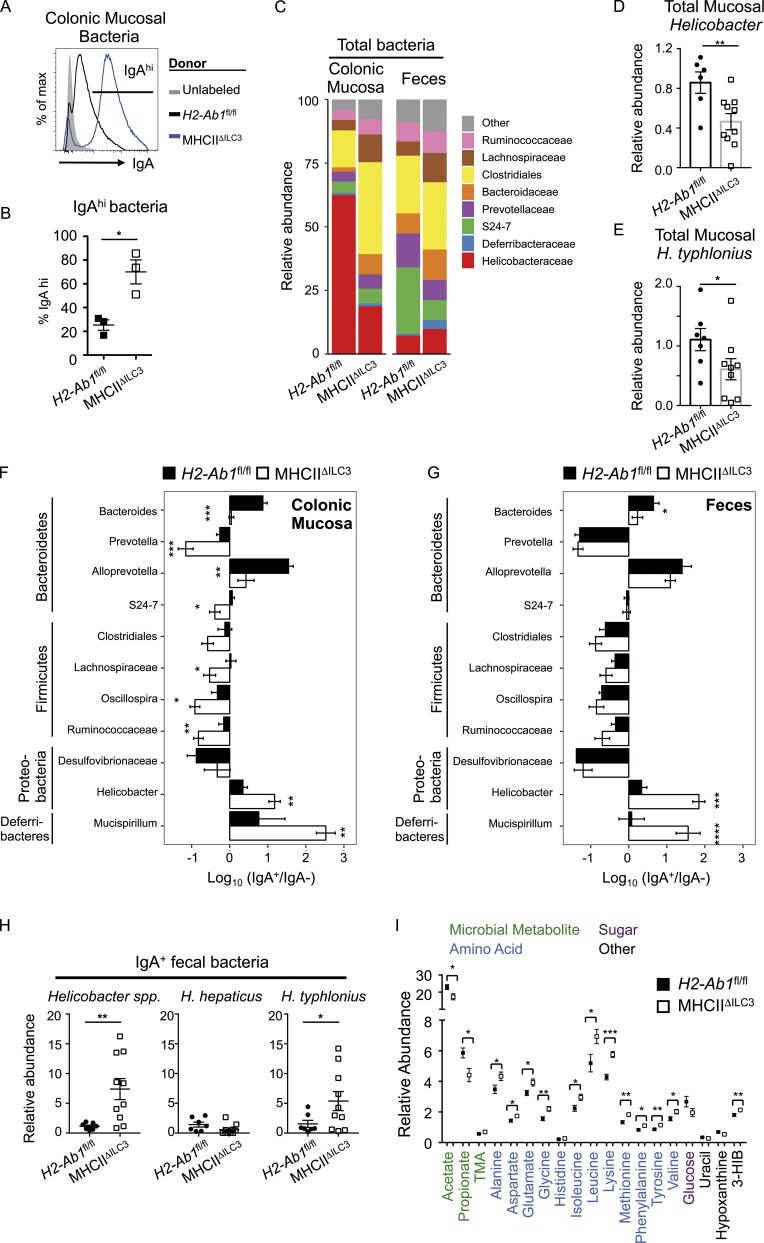
**Lack of ILC3 antigen-presentation results in dysregulated IgA coating of mucosal-dwelling bacteria in the colon.** Bacteria isolated from the colonic mucosa of IgMi mice, which lack endogenous secreted IgA, were cocultured with fecal supernatants containing free IgA. **(A and B)** Representative histogram of IgA labeling of bacteria without incubation (unlabeled; gray fill) or cultured with fecal supernatant isolated from MHCII^ΔILC3^ mice (*n* = 3, blue line) and *H2-Ab1^fl/fl^* littermate controls (*n* = 3, black line; A) and frequencies of IgA-labeled bacteria (B), representative of two independent experiments. **(C)** Quantification of total bacterial families with a relative abundance >2% in the colonic mucosa or feces. **(D and E)** Relative abundance of *Helicobacter* spp. (D) or *H. typhlonius* (E) in total colonic mucosal bacteria preparations from C. **(F and G)** IgA-seq and log10 (IgA^+^/IgA^−^) enrichment score for major taxa with an abundance of at least 1% across all samples in the colonic mucosa (F) and feces (G). Statistical analysis was performed as detailed in the Materials and methods section and additionally confirmed via Student’s *t* test. **(H)** Relative abundance of total *Helicobacter* spp., *H. hepaticus*, and *H. typhlonius* in IgA^+^ fecal bacterial samples of MHCII^ΔILC3^ and *H2-Ab1^fl/fl^* mice. **(C–H)** Analysis of MHCII^ΔILC3^ (*n* = 10) and *H2-Ab1^fl/fl^* (*n* = 7) mice, with data pooled from two independent experiments. **(I)** Relative abundance of metabolites in the feces of MHCII^ΔILC3^ (*n* = 14) and *H2-Ab1^fl/fl^* (*n* = 9) mice, representative of pooled data from three independent experiments. Metabolites selected as significant via multivariate analysis and individual Student’s *t* test comparisons performed. All data shown as mean ± SEM; all statistical analyses performed by Student’s *t* test; *P ≤ 0.05; **P ≤ 0.01; ***P ≤ 0.001; ****P ≤ 0.0001.

Increasing evidence suggests that, in contrast to innate polyreactive IgA responses, T-dependent IgA responses are driven by bacterial species that reside within the mucosal layer or attached to the epithelium itself (Palm et al., 2014; Bunker et al., 2015). To further investigate the relevance of increased IgA responses in the absence of ILC3-intrinsic antigen presentation, we isolated bacteria from the colonic mucosa or feces of mice with a mutated *IgH* locus (IgH^μγ1^; subsequently termed IgMi mice), which lack the ability to class switch and secrete antibody (Waisman et al., 2007). Thus, commensal bacteria isolated from these mice lacked endogenous IgA coating (“unlabeled”; Fig. 3 A and Fig. S3 A). Incubation of fecal bacteria from IgMi mice with fecal-derived supernatants, containing free IgA, taken from MHCII^ΔILC3^ mice and littermate controls resulted in surprisingly low levels of IgA coating (Fig. S3, A and B). In contrast, bacteria isolated from the colonic mucosa of IgMi mice exhibited a significant degree of IgA labeling following incubation (Fig. 3 A). Notably, IgA-containing supernatants from MHCII^ΔILC3^ mice resulted in a much higher level of IgA coating of colonic mucosa–associated bacteria in comparison to those incubated with IgA-containing supernatants from littermate controls (Fig. 3, A and B). To determine whether altered IgA responses in the absence of ILC3-intrinsic antigen presentation altered the composition of the commensal microbiota, 16S ribosomal RNA (rRNA) sequencing was performed on total bacteria isolated from either the feces or colonic mucosa of MHCII^ΔILC3^ mice and cohoused littermate controls (Fig. 3 C). While no significant differences were detected among major operational taxonomic units (OTUs) in fecal preparations, in line with previous findings (Hepworth et al., 2013), total bacteria populations in the colonic mucosa of MHCII^ΔILC3^ mice contained relatively less Helicobacteraceae in comparison to *H2-Ab1*^fl/fl^ controls, whereas Clostridiales were relatively enriched (Fig. 3 C). The decreased relative abundance of *Helicobacter* spp. was confirmed by quantitative PCR (qPCR) and found to be in part due to a reduced abundance of *Helicobacter typhlonius* (Fig. 3, D and E). Together these findings demonstrate that changes in microbial composition resulting from the absence of ILC3-intrinsic antigen presentation occur largely in colonic mucosa, as opposed to among luminal-dwelling species.

To determine whether alterations in colonic mucosal bacteria were driven by elevated IgA production in MHCII^ΔILC3^ mice, we performed 16S rRNA sequencing of IgA-bound bacteria (IgA-seq; Palm et al., 2014; Bunker et al., 2015). Pairwise UniFrac analysis of both colon mucosa and fecal bacterial samples from MHCII^ΔILC3^ mice and littermate controls revealed significant alterations in microbial composition, which were associated with altered IgA labeling (Fig. S3 C). Disruption of ILC3-intrinsic antigen presentation resulted in a significant enrichment in the IgA-labeling ratio of the *Helicobacter* genus as well as the Deferribacteres genus *Mucispirillum* (Fig. 3 F). Conversely, a degree of decreased IgA labeling was also observed in the colon mucosa of MHCII^ΔILC3^ mice and resulted in small but significant increases in the total relative abundance of Clostridiales and Lachnospiraceae within the colonic mucosal niche (Fig. S3 F). Differences in colonic mucosa–associated bacteria were not due to a breakdown in homeostatic containment since normal segregation of bacteria was maintained in MHCII^ΔILC3^ mice, as confirmed by fluorescent in situ hybridization (FISH) staining with a universal bacterial 16S probe (Fig. S3 D). Notably, the increased abundance of *Helicobacter* and *Mucispirillum* was seen in the IgA^+^ fraction of MHCII^ΔILC3^ mice not only in the colonic mucosa, but also in the feces (Fig. 3 G)—despite normally being poorly represented in fecal communities (Fig. S3 E)—suggesting elevated IgA labeling may favor shedding in the feces. In line with this, enrichment of *Helicobacter* was confirmed within the IgA^+^ fraction of fecal bacterial samples by qPCR and was attributed in part to increased *H. typhlonius*, but not *Helicobacter hepaticus*—a related species often used as a model of intestinal inflammation (Fig. 3 H). To determine whether changes in the mucosal-dwelling microbiota in mice lacking ILC3-intrinsic antigen presentation could have broader physiological effects, we performed ^1^H nuclear magnetic resonance (NMR) spectroscopy–based metabolic profiling on fecal samples to assess the relative metabolic profiles. Several metabolites were observed to differ between groups, including those related to gut microbial activity (Fig. 3 I). Mice lacking ILC3-intrinsic MHCII excreted lower amounts of the short chain fatty acids acetate and propionate. Moreover, several amino acids known to be metabolized by the intestinal microbiota were present in higher amounts in the feces of MHCII^ΔILC3^ mice compared with their littermate counterparts, potentially reflecting a reduction in their microbial degradation. These functional modulations in the microbiota mirrored those recently reported in mice lacking mice lacking extrathymically generated regulatory T cells—which also correlated with the reduced abundance of both *Helicobacter* and *Mucispirillum* in the intestinal mucosa (Campbell et al., 2018). Together these findings suggest antigen-presenting ILC3 act to suppress the induction of IgA responses toward colonic mucosal–dwelling bacteria in order to maintain mutualism.

### ILC3 antigen presentation regulates IgA responses to enteric pathogen infection

**Figure 4.**
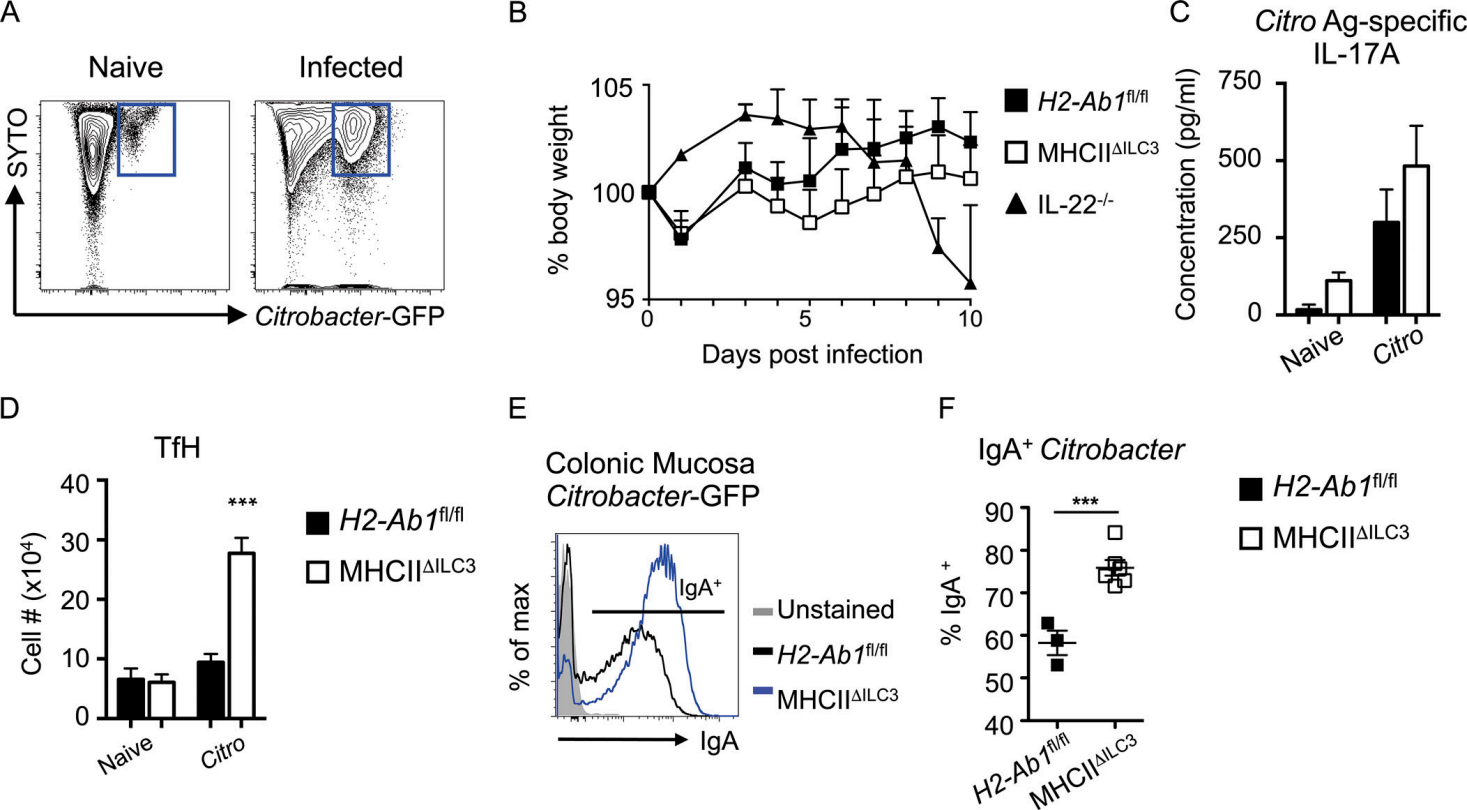
**MHCII^+^ ILC3 limit IgA responses during *C. rodentium* infection. (A)** Example flow cytometry of colonic mucosal–associated bacteria from naive mice or mice infected with *C. rodentium*–GFP. **(B–D)** Weight curves (B), antigen (Ag)-specific IL-17A production (C), and mLN TfH cell numbers (D) of *C. rodentium*–GFP-infected MHCII^ΔILC3^ (*n* = 11) and *H2-Ab1^fl/fl^* (*n* = 5) mice, representative of three independent experiments. **(E and F)** Example flow cytometry plots (E) and quantification of IgA labeling of *C. rodentium*–GFP isolated from the colonic mucosa of MHCII^ΔILC3^ (*n* = 6) and *H2-Ab1^fl/fl^* (*n* = 3) mice (F). Representative of two independent experiments. All data shown as mean ± SEM; statistical analyses by Student’s *t* test; ***P ≤ 0.001.

While our data, along with recently published studies, support the hypothesis that ILC3-intrinsic antigen presentation acts to suppress IgA labeling of mutualistic colonic mucosal–dwelling commensal bacteria species at steady state, it is notable that the same species have been attributed pathobiont potential in the context of a maladapted immune system (Honda and Littman, 2016). Moreover, the ability to establish residence within the mucus and/or epithelial niche is a characteristic also shared by pathogenic bacteria; thus, we investigated the hypothesis that antigen-presenting ILC3 could similarly play a role in limiting antibody responses to mucosal dwelling pathogens. To test this, MHCII^ΔILC3^ mice and littermate controls were infected with *Citrobacter rodentium*–enhanced GFP (eGFP), which allowed for detection of GFP^+^ bacteria in the feces and colonic mucosa of infected mice (Fig. 4 A). To limit the impact of commensal bacteria-driven phenotypes in MHCII^ΔILC3^ mice, we used 6–7-wk-old mice for infection experiments, an age at which we have previously demonstrated these mice do not yet demonstrate a notable phenotype (Hepworth et al., 2013), and which was before the age at which increases in TfH and B cells were observed (10–12 wk old; Fig. 2). Loss of ILC3-intrinsic MHCII expression had no significant effect on *C. rodentium* infection–induced body weight loss, bacterial burdens, or *C. rodentium* antigen–specific effector T helper type 17 cell responses when compared with control mice (Fig. 4, B and C; and Fig. S3 G). In line with a lack of basal commensal bacteria-driven phenotype at this time point, no difference in homeostatic mLN TfH numbers was observed between uninfected naive MHCII^ΔILC3^ mice and control littermates at this age (Fig. 4 D). In contrast, infection with *C. rodentium–*eGFP elicited a robust TfH response in MHCII^ΔILC3^ mice, which was largely lacking in control littermates (Fig. 4 D). While disruption of ILC3 antigen presentation resulted in increased numbers of IgG1 class-switched B cells, little endogenous IgG1 labeling of mucosal-associated *C. rodentium–*eGFP bacterium was seen irrespective of genotype (Fig. S3, H and I), supporting reports that IgG1 is largely generated systemically and excluded from the intestinal lumen in the absence of significant barrier disruption (Kamada et al., 2015). Critically, however, infection of MHCII^ΔILC3^ mice resulted in an increased frequency of *C. rodentium–*eGFP bacteria exhibiting labeling by IgA in the colonic mucosa and a higher intensity of IgA labeling in comparison to littermate controls (Fig. 4, E and F).

### LTi-like ILC3 limit TfH-mediated B cell class switching in an MHCII and IL-4–dependent manner

**Figure 5.**
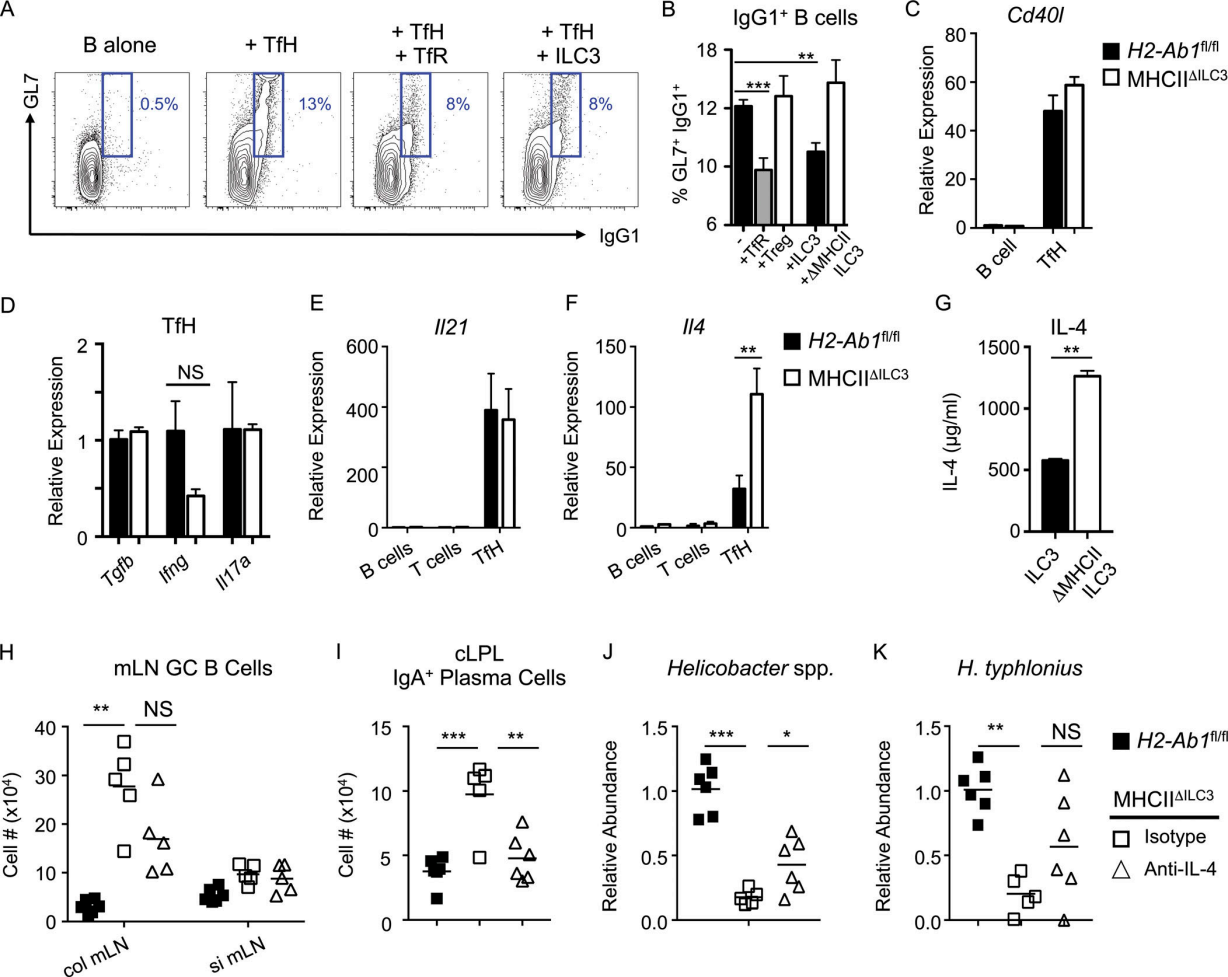
**ILC3 suppress TfH-dependent B cell class switching in an MHCII and IL-4–dependent manner.** B cells were sort-purified and cultured in vitro alone (*n* = 12), with TfH (*n* = 12) or with TfH in combination with either (CD3^+^CD4^+^/CXCR5^+^PD1^+^)CD25^+^GITR^+^ TfR (*n* = 11), (CD3^+^CD4^+^/CXCR5^−^PD1^−^)CD25^+^GITR^+^ T reg cells (*n* = 6), wild-type ILC3 (*n* = 8), or ILC3 from MHCII^ΔILC3^ mice (*n* = 6). Statistical comparisons performed by one-way ANOVA; data pooled from three independent experiments. **(A and B)** Representative flow cytometry plots (A) and frequencies (B) of (CD3^−^MHCII^+^/B220^+^CD19^+^)GL7^+^IgG1^+^ class-switched B cells. **(C–F)** Expression of *Cd40l* (C)*, Tgfb, Ifng,* and *Il17a* (D)*, Il21* (E), and *Il4* (F) in sort-purified B cells, T cells, or TfH assessed by qPCR (*n* = 3 per group), representative of two independent experiments. Statistical comparisons performed by Student’s *t* test. **(G)** Concentration of IL-4 protein in supernatants from cocultures containing B cells and TfH cultured with wild-type of MHCII-deficient ILC3, as in A and B. Statistical comparisons performed by Student’s *t* test. **(H and I)** Cell numbers of mLN (CD3^−^MHCII^+^/B220^+^ CD19^+^)GL7^+^Fas^+^ GC B cells (H) and (CD3^−^MHCII^+^)B220^−^IgA^+^ plasma cells in the cLPL of anti–IL-4 (*n* = 5) or isotype (*n* = 5) control–treated MHCII^ΔILC3^ and *H2-Ab1^fl/fl^* (*n* = 6) mice (I). **(J and K)** Relative abundance of *Helicobacter* spp. (J) and *H. typhlonius* (K) in total colonic mucosal bacteria preparations from anti–IL-4 or isotype control–treated MHCII^ΔILC3^ and *H2-Ab1^fl/fl^* mice. **(H–K)** Statistical comparisons performed by one-way ANOVA; data representative of two independent experiments. All data shown as mean ± SEM; * P ≤ 0.05, **P ≤ 0.01; ***P ≤ 0.001.

To determine whether antigen presentation by LTi-like ILC3 directly modulates TfH function and their ability to stimulate B cell class switching, we cocultured TfH and B cells isolated from mice immunized with 4-hydroxy-3-nitrophenylacetyl (NP)–OVA in CFA, as previously described (Sage and Sharpe, 2015; Sage et al., 2016). Coculture with TfH induced a population of IgG1^+^GL7^+^ class-switched B cells, which could be suppressed by the addition of TfR, as previously described (Sage et al., 2016; Fig. 5 A). Similarly, addition of ILC3 to TfH–B cell cocultures resulted in reduced frequencies of class-switched B cells and was dependent on antigen presentation via MHCII (Fig. 5, A and B). While this assay did not allow for modeling of mucosal IgA class switching, these data nonetheless successfully provided proof of concept that ILC3 directly modulate TfH-driven class switching in vitro via antigen presentation.

To further investigate how TfH function is modulated in the absence of ILC3 regulation in vivo, we sort-purified TfH from MHCII^ΔILC3^ mice and littermate controls. We were unable to find any significant differences in TfH provision of CD40L (Fig. 5 C) or expression of *Tgfb*, *Ifng*, or *Il17a* in the absence of ILC3-intrinsic MHCII (Fig. 5 D). Recent evidence suggests that while initial TfH interactions with B cells are driven by production of IL-21 to select high-affinity B cell clones, progression of GC reactions and maturation of plasma cells requires a switch in TfH cytokine production toward a predominantly IL-4–producing phenotype (Weinstein et al., 2016). To investigate whether ILC3 antigen presentation acts as a checkpoint in TfH progression and cytokine production, we first analyzed IL-21 and IL-4 mRNA levels in sort-purified TfH from the mLN by qPCR. While TfH isolated from MHCII^ΔILC3^ mice expressed comparable levels of IL-21 mRNA to their littermate controls, IL-4 message was significantly higher in the absence of ILC3-intrinsic antigen presentation (Fig. 5, E and F). Similarly, MHCII-deficient ILC3 cocultured with TfH induced more IL-4 protein in culture supernatants when compared with MHCII^+^ ILC3 (Fig. 5 G). To determine whether increased IL-4 production by mLN-resident TfH in the absence of ILC3 antigen presentation contributed to elevated GC B cell responses and IgA responses against mucosal-dwelling commensals, MHCII^ΔILC3^ mice were treated with anti–IL-4 neutralizing antibody or an isotype control from 6 wk of age and compared with *H2-Ab1*^fl/fl^ littermates. Strikingly, while isotype control–treated mice developed increased GC B cell responses in the colon-draining mLN and elevated numbers of IgA^+^ plasma cells, mice treated with anti–IL-4 developed a markedly suppressed GC response in the mLN and had significantly reduced numbers of IgA^+^ plasma cells in the colon (Fig. 5, H and I). Moreover, mice treated with anti–IL-4 antibody retained significantly higher levels of mucosal-associated *Helicobacter* in comparison to isotype-treated control mice, including higher levels of *H. typhlonius*—although this did not reach statistical significance (Fig. 5, J and K).

Collectively these findings suggest localization of antigen-presenting LTi-like ILC3 within the colon-draining lymph node facilitates interactions with TfH and the subsequent regulation of mucosal IgA responses toward the commensal microbiota, particularly species that establish residence in “border niches” such as the mucosa or epithelium. Our data suggest that common pathways that orchestrate the localization and interactions of multiple cell types within the interfollicular niche of the mLN—including TfH, B cells, and ILC3—may have important roles in orchestrating IgA responses and mutualism with the commensal microbiota to maintain intestinal health. Interestingly, polymorphisms in *Gpr183*, *Cxcr5*, and *Ccr6* have all been identified as risk alleles for inflammatory bowel disease (Jostins et al., 2012), suggesting aberrant positioning of immune cells, including ILC3, could predispose to intestinal disease—although our findings indicate only *Gpr183* is absolutely required for ILC3 localization within the mLN. Of note, *Gpr183*-dependent positioning of MHCII^+^ ILC3 at the interface between the T cell zone and B cell follicle overlaps with the recently described distribution of mLN stromal cells expressing *Ch25h*—the key enzyme for generation of the *Gpr183* ligand 7α,25-hydroxycholesterol—suggesting chemokine gradients orchestrated by defined stromal populations determine positioning of ILC3 in both the colon and the lymph node, in line with recent publications (Chu et al., 2018; Emgård et al., 2018; Rodda et al., 2018). Intriguingly, ILC2 have also been shown to localize to this interfollicular niche and also have the capacity to express both MHCII as well as costimulatory molecules that may endow the cells with the ability to modulate TfH responses (Oliphant et al., 2014; Schwartz et al., 2017; Halim et al., 2018), while ILC2 also produce soluble factors that can positively regulate B cell responses (Moro et al., 2010; Jackson-Jones et al., 2016). Further studies are required to determine whether ILC3 and ILC2 antagonistically modulate TfH and B cell responses in health and disease.

Our findings further implicate interactions with ILC3 and TfH in the intestinal draining lymph node as key regulators of intestinal B cells and IgA responses toward mucosal bacteria. However, it should be noted that we also observed formation of B cell–containing isolated lymphoid follicles in the colon (Fig. 2 M) and, while we found no evidence for TfH responses locally in the tissue, we cannot rule out secondary interactions between ILC3 and adaptive immune cells in the colon itself. Interactions between the host and the microbiota are dynamic, and both innate and T cell–dependent IgA have been shown to be critical in regulating intestinal bacterial mutualism and for selecting and maintaining refined communities of bacteria with beneficial properties (Fagarasan et al., 2010; Sutherland et al., 2016). Recent studies have demonstrated that the majority of IgA is induced within the small intestinal tract in a T cell–independent manner and is polyreactive (Bunker et al., 2017). However, in this study, the absence of ILC3-intrinsic MHCII had no effect on TfH and IgA responses in the small intestine–draining lymph node, small intestinal lamina propria, and associated Peyer’s patches. In contrast, we observed a marked regulation of TfH and GC B cells in the colon-draining mLN, which further correlated with the numbers of IgA-producing plasma cells in the colon lamina propria and IgA-labeling of colonic mucosal–dwelling bacteria. The reasons for this tissue compartmentalization and specificity are unclear, but it is notable that the distribution and MHCII expression of ILC3 differs in these tissues (Fig. S1), as does microbial load, while studies to elucidate how and where ILC3 acquire commensal bacteria-derived antigen will likely be highly informative in dissecting this immunological cross-talk further. ILC3 regulated TfH responses through MHCII-dependent suppression of IL-4, thus preventing the elevated GC responses and IgA production that develop in the absence of ILC3 antigen presentation. Further studies are required to determine the precise nature of ILC3 interactions with TfH and the full consequences of this interaction on TfH function—in particular whether antigen-presenting ILC3 use additional costimulatory or coinhibitory signals to modulate TfH survival, proliferation, or function.

Increased T cell–dependent IgA coating has previously been suggested to preferentially target bacteria that reside close to the host epithelial layer or within the mucus layer, which may be inherently more immunostimulatory or which could possess an increased potential to drive intestinal inflammation in the context of a maladapted immune system or inflammation (Palm et al., 2014; Bunker et al., 2015). These findings provoke the question as to why ILC3 would limit homeostatic IgA responses to mucosal-resident bacterial species, which may have pathobiont potential. However, while *Helicobacter* species have been demonstrated to act as pathobionts in the context of a maladapted immune system, it is of note that *Helicobacter* primarily induce regulatory responses in healthy immunocompetent mice (Kullberg et al., 2002, 2006; Chai et al., 2017; Danne et al., 2017; Xu et al., 2018). Furthermore, one recent study elegantly demonstrated a mutualistic role for both *Helicobacter* and *Mucispirillum* colonization of the mucosa early in life (Campbell et al., 2018). Notably, in this study failure of these bacterial species to colonize in the absence of regulatory T cells was associated with a dysregulated metabolome and affected host growth. Strikingly, comparable changes in intestinal metabolites were also found in this study, where mucosal-dwelling species were also lost from this colonic niche as a consequence of elevated IgA responses in the absence of ILC3 regulation. Together this suggests mucosal-dwelling bacteria, including *Helicobacter*, may normally confer benefits to the host, despite their pathobiont potential, and that ILC3 act as a checkpoint to control the quality and magnitude of T-dependent IgA in order to support mutualism with the mucosal-dwelling microbiota.

## Materials and methods

### Mice

Age- and sex-matched C57BL/6 mice were purchased from Envigo Laboratories. *Id2*^CreERT2^ mice were originally purchased from The Jackson Laboratory. ROSA-26^RFP^ mice were a gift from Hans-Joerg Fehling (University Clinics, Ulm, Germany). CXCR5^flox/flox^ mice were generated as previously described (Bradford et al., 2017). *Rorc*^Cre^ x *H2-Ab1*^flox/flox^ (MHCII^ΔILC3^) and *H2-Ab1*^flox/flox^ littermate controls were generated as previously described (Hepworth et al., 2013), and generated through breeding of MHCII^ΔILC3^ males with *H2-Ab1*^flox/flox^ females to control for vertical transmission. Experimental littermate progeny were subsequently cohoused with random distribution of genotypes among cages. *Rorc*^Cre^ × *Gpr183*^flox/flox^ mice were generated as recently described (Emgård et al., 2018), and maintained at the Karolinska Institute, Stockholm, Sweden. RORγt^eGFP^ mice were originally a gift from Gerard Eberl (Pasteur Institute, Paris, France), and IgMi mice were originally a gift from Ari Waisman (IMB Mainz, Mainz, Germany). All transgenic mouse strains were used as cohoused littermates and maintained under specific pathogen free conditions at the University of Manchester, Manchester, UK, unless otherwise specified. All animal experiments were performed under license of the UK Home Office and under approved protocols at the University of Manchester. Experiments performed at the Karolinska Institute were performed under the approval of the Linköping Animal Experimentation Ethics Committee.

### In vivo IL-4 neutralization

Mice were administered 200 µg/ml anti–IL-4 mAb (clone 11B11; BioXcell) twice weekly (every 3–4 d) for 4 wk. Control group was treated with 200 µg/ml monoclonal IgG1 isotype control (clone TNP6A7; BioXcell).

### Tissue processing

mLN and spleen were processed by passing them through a 70-µm nylon filter, and red blood lysis was performed where necessary. For intestinal lamina propria lymphocyte preparations, intestinal tissue was isolated, associated fat and Peyer’s patches removed, and tissues cut open longitudinally. Luminal contents were removed by shaking in cold PBS. Epithelial cells and intra-epithelial lymphocytes were removed by shaking tissues in stripping buffer (1 mM EDTA, 1 mM dithiothreitol, and 5% FCS) for two rounds of 20 min at 37°C. Lamina propria lymphocytes were isolated by digesting the remaining tissue in 1 mg/ml collagenase/dispase (Roche) and 20 µg/ml DNase I (Sigma-Aldrich) for 45 min at 37°C. Liberated cells were then extracted by passing the tissue and supernatant over a 70-µm nylon filter and centrifugation to isolate lamina propria lymphocytes.

### Flow cytometry

Single-cell preparations were stained with antibodies to the following markers: anti-CD25 (clone PC61.5; eBioscience), anti-CD3 (clone 145-2C11; eBioscience), anti-CD5 (clone 53–7.3; eBioscience), anti-NK1.1 (clone PK136; eBioscience), anti-GITR (DTA-1; BioLegend), anti-CD90.2 (clone 30-H12; BioLegend), anti-B220 (clone RA3-6B2; eBioscience), anti-CD11b (clone M1/70; eBioscience), anti-CD11c (clone N418; eBioscience), anti-MHCII (clone M5/114.15.2; eBioscience), anti-CD45 (clone 30-F11; BioLegend), anti-CD4 (clone GK1.5; BD), anti-ICOS (clone 15F9; BioLegend), anti-CXCR5 (L138D7; BioLegend), anti-PD-1 (clone RMP1-30; BioLegend), anti-IgG1 (clone RMG1-1; BioLegend), anti-Fas (clone 15A7; eBioscience), anti-GL7 (clone GL7; BioLegend), anti-CD38 (clone 90; eBioscience), anti-CD19 (clone 1D3; BD), anti-IgA (clone mA-6E1; eBioscience), anti-IgD (clone 11-26c.2a; BioLegend), anti-CD138 (clone 281–2; BioLegend), anti-CCR6 (clone 29-2L17; eBioscience; or clone 140706; R&D Systems), anti-CD127 (clone A7R34; eBioscience), anti-CD90.2 (clone 30-H12; BioLegend), anti-KLRG-1 (clone 2F1; eBioscience), anti-NKp46 (clone 29A1.4; eBioscience), biotin anti-CXCR5 (clone 2G8; BD), and biotin anti-ST2 (clone RMST2-33; eBioscience). Dead cells were excluded from analysis using the LIVE/DEAD Fixable Aqua Dead Cell Stain (Life Technologies). Intracellular staining was performed using the FoxP3 Transcription Factor Buffer set (eBioscience), and cells were stained for anti-FoxP3 (FJK-16s; eBioscience), anti-RORγt (clone B2D; eBioscience), and anti-Bcl6 (clone 7D1; BioLegend). For LTα_1_β_2_ detection, cells were blocked with donkey anti-mouse IgG Fab fragments (Jackson ImmunoResearch) and then stained with recombinant mouse LTβR Fc chimera (R&D Systems). Cells were stained with biotin-conjugated donkey anti-mouse IgG (Jackson ImmunoResearch) followed by staining with antibodies and APC-conjugated streptavidin (eBioscience).

### Bacterial flow cytometry

Feces were collected in Fast Prep lysing Matrix A tubes (MP Biomedicals), resuspended in 1 ml of PBS per 100 mg fecal material, and incubated at 4°C for 20 min. Colon mucosal samples were isolated by removing feces and loose material associated with the colon tissue and scraping along the length of a colon with a cell scraper (Greiner Bio One) into fresh cold PBS. Bacterial suspensions were resuspended in a final volume of 2 ml PBS and incubated at 4°C for 20 min. Samples were homogenized in a FastPrep-24 Tissue homogenizer (MP Biomedicals) for 30 s. After homogenization, samples were centrifuged at 50 × *g* for 15 min at 4°C to remove debris and the bacteria-containing supernatant transferred through 70 µm filters into a new tube. Bacteria were washed in FACS buffer (PBS, 2% FCS, and 5 mM EDTA) and pelleted at 8,000 × *g* for 5 min. For flow cytometry, bacterial pellets were resuspended in 100 µl FACs buffer containing SYTO 9 green fluorescent nucleic stain (10 µM; Life Technologies), incubated at 4°C for 15 min, and subsequently stained with 1 µg/ml of an anti-mouse IgA-PE antibody (eBioscience) for 30 min at 4°C. Samples were thoroughly washed before acquisition on the flow cytometer (BD Fortessa).

### IgA-seq and 16S rRNA sequencing

Fecal or colonic mucosa–associated bacteria were isolated as detailed above and sorted by magnetic-activated cell sorting (MACS)to separate IgA^+^ and IgA^−^ bacterial fractions. Samples were stained with anti-IgA PE antibody as above, washed, and incubated in staining buffer with anti-PE MACS beads (Miltenyi Biotec) as per the manufacturer’s instructions for 30 min at 4°C. An aliquot of total bacteria was centrifuged before IgA sorting for 16S rRNA sequencing analysis. The remaining stained sample was washed and separated by passing samples over LS-MACS columns (Miltenyi Biotec). The negative, unbound fraction (IgA^−^) was extracted, while the bound IgA^+^ fraction was further purified by a second round of column separation. Samples were centrifuged and stored at 20°C for further 16S sequencing analysis. Post-magnetic bead–enriched samples were analyzed for purity by flow cytometry; positive fractions were ≥85% IgA^+^, and negative fractions were typically <5% IgA^+^.

Bacterial DNA from total and IgA-sorted fecal and colonic bacteria was isolated using the PowerSoil DNA Isolation Kit (Cambio Limited) according to the manufacturer’s instructions. Pre-amplification of the V3V4 region of 16S rRNA was performed by PCR in triplicate using Phusion Hot Start II High Fidelity Polymerase (Thermo Fisher Scientific) using previously published primer pairs containing adaptor sequences for downsteam use on Illumina platforms. 16S sequencing was performed by using the Illumina MiSeq platform, and initial processing and quality assessment of the sequencing data were performed at the Centre for Genomics, University of Liverpool, Liverpool, UK, using an in-house pipeline.

### 16S rRNA sequencing analysis

16S rRNA sequencing was undertaken at the Centre for Genomic Research, University of Liverpool. Analysis of IgA-seq data were performed at the Children’s Hospital of Philadelphia Microbiome Center, Philadelphia, PA. Sequence data (NCBI BioProject ID: PRJNA518140) were processed using QIIME version 1.9. Read pairs were joined to form a complete V3V4 amplicon based on a minimum overlap of 35 bases and maximum overlap difference of 15%. The reads were filtered based on a quality threshold of Q20. OTUs were selected by clustering reads at 97% sequence similarity using UCLUST v. 1.2.22. Taxonomic assignments were generated by comparing the representative OTU sequences to the Greengenes reference database v. 13_8. A phylogenetic tree was inferred from the OTU data using FastTree2. Similarity between samples was assessed by weighted and unweighted UniFrac distance. For statistical analysis, data files from QIIME were analyzed in the R environment. Weighted and unweighted UniFrac distances were used to visualize global differences in bacterial community compositions using Principal Coordinates Analysis. Community-level differences between genotypes were assessed using the PERMANOVA test for each sample type and IgA status. Pairwise weighted and unweighted distances between IgA^+^ and IgA^−^ samples for each mouse were tested with linear mixed effects models for difference between the genotypes. A linear mixed effects model was fit on the log10 (IgA^+^/IgA^−^) values to compare the magnitude of IgA response in MHCII^ΔILC3^ and *H2-Ab1*^flox/flox^ control mice. Only the taxa with a mean abundance of greater than 1% across samples were used for the tests. Multiple tests were corrected for false discovery rate using Benjamini-Hochberg method.

### Real-time PCR

Total RNA was purified using the RNeasy Micro Kit (Qiagen), and cDNA was prepared using the high-capacity cDNA reverse transcription kit (Applied Biosystems). Real-time qPCR was performed with the real-time PCR StepOnePlus system (Applied Biosystems), using LightCycler 480 SYBR Green I Master Mix (Roche) and with the following primers: *Ebi2* forward, 5′-ACA​ACG​GAG​GTC​CTA​GCC​A-3′; *Ebi2* reverse, 5′-GCT​GTG​GTG​GGC​ATA​GAG​A-3′; *Cxcr5* forward, 5′-GAC​CTT​CAA​CCG​TGC​CTT​TCT​C-3′; *Cxcr5* reverse, 5′-GAA​CTT​GCC​CTC​AGT​CTG​TAA​TCC-3′; *Bcl6* forward, 5′-CCT​GTG​AAA​TCT​GTG​GCA​CTC​G-3′; *Bcl6* reverse, 5′-CGC​AGT​TGG​CTT​TTG​TGA​CG-3′; *Dll1* forward, 5′-CTA​TGG​CAA​GGT​CTG​TGA​GCT​G-3′; *Dll1* reverse, 5′-ATC​TGA​ACA​TCG​TCC​TCC​ATT​G-3′; *Tnfsf13* forward, 5′-ACC​CAG​AAG​CAC​AAG​AAG​AAG​C-3′; *Tnfsf13* reverse, 5′-GTA​CTG​GTT​GCC​ACA​TCA​CCT​C-3′; *Il4* forward, 5′-GAG​AGA​TCA​TCG​GCA​TTT​TGA-3′; *Il4* reverse, 5′-TCT​GTG​GTG​TTC​TTC​GTT​GC-3′; *Cd40l* 5′-GTG​AGG​AGA​TGA​GAA​GGC​AA-3′; *Cd40l* reverse, 5′-CAC​TGT​AGA​ACG​GAT​GCT​GC-3′; *Ifng* forward, 5′-GGA​GGA​ACT​GGC​AAA​AGG​AT-3′; *Ifng* reverse, 5′-TTC​AAG​ACT​TCA​AAG​AGT​CTG​AGG-3′; *Il17a* forward, 5′-TGT​GAA​GGT​CAA​CCT​CAA​AGT​C-3′; *Il17a* reverse, 5′-AGG​GAT​ATC​TAT​CAG​GGT​CTT​CAT​T-3′; *Tgfb1* forward, 5′-CTG​GGC​ACC​ATC​CAT​GAC-3′; *Tgfb1* reverse, 5′-CAG​TTC​TTC​TCT​GTG​GAG​CTG​A-3′; *bactin* forward, 5′-TCC​TAT​GTG​GGT​GAC​GAG-3′ and *bactin* reverse, 5′-CTC​ATT​GTA​GAA​GGT​GTG​GTG-3′. Real-time PCR was performed according to the Standard Thermal Cycler Protocol. Samples were incubated for 95°C for 10 min, followed by denaturation for 15 s at 95°C and combined annealing/extension for 1 min at 60°C for a total of 40 cycles. Data were normalized to the housekeeping gene (*bactin*), analyzed using the 2–ΔΔCT method, and expressed relative to control groups as indicated. TaqMan Gene Expression Assays were additionally used for assessment of *Il21* (Mm00517640_m1) and *hprt* (Mm03024075_m1) expression (Applied Biosystems).

### Bacterial 16S qPCR

Bacterial DNA samples were amplified using the following primers: total 16S forward, 5′-ACT​CCT​ACG​GGA​GGC​AGC​AGT-3′; total 16S reverse, 5′-ATT​ACC​GCG​GCT​GCT​GGC-3′; total *Helicobacter* forward, 5′-GCT​ATG​ACG​GGT​ATC​C-3′; total *Helicobacter* reverse, 5′-GAT​TTT​ACC​CCT​ACA​CCA-3′; *H. typhlonius* forward, 5′-AGG​GAC​TCT​TAA​ATA​TGC​TCC​TAG​AGT-3′; *H. typhlonius* reverse, 5′-ATT​CAT​CGT​GTT​TGA​ATG​CGT​CAA-3′; *H. hepaticu*s forward, 5′-GCA​TTT​GAA​ACT​GTT​ACT​CTG-3′; *H. hepaticus* reverse, 5′-CTG​TTT​TCA​AGC​TCC​CCG​AAG-3′. Real-time qPCR was performed with the real-time PCR StepOnePlus system (Applied Biosystems) using the reagents and qPCR conditions stated above. Data were normalized to the total bacteria (16S), analyzed using the 2–ΔΔCT method, and expressed relative to control groups as indicated.

### Intestinal metabolite analysis

The metabolic profiles of fecal samples were measured using ^1^H NMR spectroscopy as previously described (Beckonert et al., 2007). Briefly, fecal samples (30 mg) were defrosted and combined with 600 µl of water and zirconium beads (0.45 g). Samples were homogenized with a Precellys 24 instrument (45 s per cycle, speed 6,500, two cycles) and spun at 14,000 × *g* for 10 min. The supernatants (400 µl) were combined with 250 µl phosphate buffer (pH 7.4, 100% D_2_O, 3 mM NaN_3_, and 1 mM of 3-(trimethyl-silyl)-[2,2,3,3-^2^H4]-propionic acid [TSP] for the chemical shift reference at δ 0.0) before centrifugation at 14,000 × *g* for 10 min, and then transferred to 5 mm NMR tubes for analysis on a Bruker 600 MHz spectrometer operating at 300 K. ^1^H NMR spectra were acquired for each sample using a standard one-dimensional pulse sequence using the first increment of the nuclear overhauser effect pulse sequence for water suppression as previously described (Beckonert et al., 2007). Raw spectra were automatically phased, baseline-corrected, and calibrated to TSP using Topspin 3.2 (Bruker Biospin) and then digitized in a MATLAB environment (Version 2018; MathWorks Inc.) using in-house scripts. Redundant spectral regions (related to water and TSP resonance) were removed, and the spectral data were manually aligned and normalized to the probabilistic quotient using in-house MATLAB scripts. The peak integrals (relating to relative abundance) for discriminatory metabolites were calculated for each sample.

### ELISA

Mouse fecal IgA titers were measured using the Mouse IgA ELISA Quantitation Set (Bethyl Laboratories) following the manufacturer’s instructions. Mouse fecal albumin was measured using the Mouse Albumin ELISA Quantitation Set (Bethyl Laboratories) following the manufacturer’s instructions. For both ELISAs, fecal samples were diluted 1/100 or 1/1,000, and concentration was determined based on a standard curve. For measurement of *C. rodentium*–specific antibodies in serum or fecal supernatants by ELISA, 10 µg/ml *C. rodentium* antigen was coated on 96-well plates, and sera were incubated in doubling dilutions. Antigen-specific IgG and antigen-specific IgA were detected using an anti-mouse IgG-HRP antibody (BD Biosciences) and an anti-mouse IgA-HRP (Bethyl Laboratories). For measurement of mouse IL-4 in cell culture, a capture anti-mouse IL-4 antibody (clone 11B11; donated by A. MacDonald, University of Manchester, Manchester, UK), recombinant mouse IL-4 (BioLegend) and a detection biotinylated anti-mouse IL-4 antibody (clone 24G2; BioLegend) were used. The mouse IL17A ELISA kit (Invitrogen) were used to detect cytokines in cells stimulated with *C. rodentium* antigen. Plates were developed with TMB peroxidase substrate (BD Biosciences), and optical densities were measured using a plate spectrophotometer.

### *C. rodentium* infection

*C. rodentium*–eGFP has previously been described (Bergstrom et al., 2010). Mice were infected with 10^9^ CFUs of bacteria per oral gavage, and bacterial burdens were measured in the feces by plating serial dilutions on MacConkey agar plates and incubating overnight at 37°C to quantify CFU per gram of fecal material.

### In vitro coculture assay

Cocultures of ILC3, TfH, and B cells were performed using a previously described method (Sage and Sharpe, 2015), with minor alterations. Briefly, mice were subcutaneously immunized in the flank with 50 µg NP-OVA in CFA. 1 wk following immunization, inguinal lymph nodes were harvested and enriched for B and T cell populations by magnetic bead separation (Miltenyi Biotec) following the manufacturer’s instructions. Enriched populations were further sort-purified to >98% purity as live CD45^+^CD19^+^B220^+^ (B cells), or as live CD45^+^CD3^+^CD4^+^ICOS^+^CXCR5^+^GITR^−^ TfH. LTi-like ILC3 were sort-purified from naive mesenteric and inguinal lymph nodes following preenrichment to deplete B cells, as above, as CD45^+^Lineage^−^CD127^+^CD90^+/int^KLRG1^−^CCR6^+^. Combinations of B cells, TfH, and ILC3 were cultured in a ratio of 50,000:30,000:15,000 in the presence or absence of 2 µg/ml anti-CD3, 5 µg/ml anti-IgM, and 20 µg/ml NP-OVA. Following 6 d of culture, cells were harvested and stained for CD19, B220, GL7, CD38, and IgG1 to quantify B cell class switching.

### Immunofluorescence imaging

Colon tissue was fixed in 1–2% paraformaldehyde (Sigma-Aldrich) for 3 h at 4°C, washed three times in PBS for 10 min, dehydrated in a 10%→20%→30% sucrose gradient, and frozen in optimal cutting temperature medium. 7-µm sections were cut and collected onto Superfrost Plus slides (VWR). For staining, samples were hydrated in 0.5% BSA/PBS for 10 min and incubated directly with B220 (RA3-6B2)-APC and GL7-FITC (1/200 dilution). Slides were washed for 3 min in 0.5% BSA/PBS, stained for 10 min with DAPI, and mounted with ProLong Gold (Invitrogen). Slides were visualized under a fluorescence microscope (Zeiss) or a slide scanner microscope (Olympus), and images were processed with ImageJ. Sections of mLN were prepared and stained for RORγt and CD3 expression as previously described (Mackley et al., 2015). Briefly, 7-µm-thick sections of tissue were cut, fixed in cold acetone at 4°C for 20 min, and then stored at −20°C before staining. Antibodies raised against the following mouse antigens were used: CD3 (1:50; clone eBio500A2; eBioscience), IL-7Rα (1:25; clone A7R34; eBioscience), and RORγt (1:30; clone AFKJS-9; eBioscience). Detection of RORγt was achieved through amplification of the primary signal as described previously (Kim et al., 2011). Briefly, purified rat primary antibodies against RORγt were detected with donkey anti-rat-IgG-FITC (1:150; Jackson ImmunoResearch), and subsequently rabbit anti-FITC-AF488 (1:200; Life Technologies) and donkey anti-rabbit-IgG-AF488 (1:200; Life Technologies). Biotinylated anti-CD3 antibodies were detected with SA-AF555 (1:500; Life Technologies). Expression of MHCII was detected using anti-mouse I-A/I-E antibody conjugated to Alexa Fluor 647 (clone M5/114.15.2; BioLegend), as previously demonstrated (Hepworth et al., 2015). Sections were counterstained with DAPI (Invitrogen) and mounted using ProLong Gold (Invitrogen). Image acquisition and analysis were performed with a Zeiss 780 Zen microscope using Zen software.

### FISH staining

For FISH, the colon was fixed in Carnoy’s fixative (60% methanol, 30% chloroform, and 10% acetic acid) overnight, washed in methanol for 30 min, and then stored in absolute ethanol until embedded in paraffin. Longitudinal sections (5 µm) were hybridized to a bacterial 16S rRNA gene probe: [Cy3] GCTGCCTCCCGTAGGAGT (Eurofins Genomics). Sections were counterstained with DAPI (Invitrogen) and mounted using ProLong Gold (Invitrogen). Slides were visualized under a fluorescence microscope (Zeiss) and images processed with ImageJ.

### Statistical analyses

Results represent mean ± SEM. Statistical analyses were performed by Student’s *t* test, Mann–Whitney *U* test, or one-way ANOVA unless specified otherwise.

### Online supplemental material

Fig. S1 shows orchestration of ILC3 localization in the mLN. Fig. S2 shows that antigen-presenting ILC3 limit TfH and B cell responses. Fig. S3 shows dysregulated IgA coating of commensal bacteria and responses to enteric pathogens in the absence of ILC3 antigen presentation.
